# Use of Influenza Risk Assessment Tool for Prepandemic Preparedness

**DOI:** 10.3201/eid2403.171852

**Published:** 2018-03

**Authors:** Stephen A. Burke, Susan C. Trock

**Affiliations:** Centers for Disease Control and Prevention, Atlanta, Georgia, USA (S.C. Trock, S.A. Burke); Battelle, Atlanta (S.A. Burke)

**Keywords:** pandemic influenza, pandemic preparedness, influenza risk assessment, viruses, Influenza Risk Assessment Tool, respiratory infections, bioterrorism and preparedness

## Abstract

In 2010, the Centers for Disease Control and Prevention began to develop an Influenza Risk Assessment Tool (IRAT) to methodically capture and assess information relating to influenza A viruses not currently circulating among humans. The IRAT uses a multiattribute, additive model to generate a summary risk score for each virus. Although the IRAT is not intended to predict the next pandemic influenza A virus, it has provided input into prepandemic preparedness decisions.

Planning and preparation for influenza pandemics are major challenges to public health authorities for many reasons, not the least of which is the inherent variability and unpredictability of the influenza virus (*1*). Just in the past decade, infections from multiple new influenza viruses have occurred in humans, representing influenza A subtypes such as H1N2, H3N2v, H5N1, H5N6, H6N1, H7N2, H7N3, H7N7, H7N9, H9N2, and H10N8. In response to these findings, prepandemic vaccines were developed for some of these viruses (*2*–*5*). In 2009, a new virus, subsequently designated influenza A(H1N1)pdm09, emerged in humans in North America and quickly spread, causing the first influenza pandemic of the 21st century (*6*). Although only 3 hemagglutinin (HA) subtypes of influenza (H1, H2, and H3) are known to have caused human pandemics (*7*), the emergence and spread of influenza A(H5N1) and, more recently, influenza A(H7N9), with associated high death rates in humans, are of great concern. If these or other influenza A viruses not currently circulating among humans develop the capability to transmit efficiently among humans, they pose a risk for causing a pandemic that could be associated with high rates of illness and death (*8*,*9*).

The task of risk mitigation planning and preparedness for pandemic influenza is difficult, and a tool is needed that systematically evaluates different influenza viruses to inform decisions related to the prioritization and allocation of resources for vaccine development, influenza surveillance strategies, and research initiatives. In this context, the Centers for Disease Control and Prevention (CDC; Atlanta, GA, USA) developed the Influenza Risk Assessment Tool (IRAT) with the goal to systematically evaluate influenza A viruses that are not circulating in humans but potentially pose a pandemic risk (*10*).

The IRAT uses a common decision analysis approach that incorporates input from multiple elements or attributes, applies a weighting scheme, and generates a score to compare various options or decisions (*11*). In regard to the evaluation of animal-origin influenza viruses for their potential human pandemic risk, 2 specific questions were developed related to the potential risk for emergence and consequent potential impact: 1) What is the risk that a virus not currently circulating in humans has the potential for sustained human-to-human transmission? (emergence question); and 2) If a virus were to achieve sustained human-to-human transmission, what is the risk that a virus not currently circulating among humans has the potential for substantial impact on public health? (impact question).

In developing the IRAT, a working group of international influenza experts in influenza virology, animal health, human health, and epidemiology identified 10 risk elements and definitions. These elements were described previously (*10*); in brief, they include virus properties (genomic variation, receptor-binding properties, transmissibility in animal models, and antiviral treatment susceptibility) and host properties (population immunity, disease severity, and antigenic relationship to vaccines). The final 3 elements are based on the epidemiologic and ecologic evidence: infection in humans, infections in animals, and global distribution in animals. These elements are used to answer the 2 risk questions to evaluate an influenza virus of interest. The 10 elements are ranked and weighted on the basis of their perceived importance to answering the specific risk questions and an aggregate risk score is generated.

Since its inception, the IRAT has facilitated the evaluation of multiple viruses and contributed information to decisions related to US pandemic planning, such as selection of candidate vaccine viruses (CVVs) and vaccines for the Strategic National Stockpile of prepandemic influenza vaccines (*12*). We summarize the evaluation of 14 animal viruses and discuss the strengths and limitations of the IRAT as a tool supporting CDC’s Preparedness and Response Framework for Influenza Pandemics (*13*), a document that outlines key public health decisions and actions to be taken at specific times during an influenza pandemic.

## Methods

### Scoring Procedure

The ranking and weighting of risk elements used to answer the 2 standard IRAT risk questions (emergence, impact) was predetermined by the working group of international influenza experts (*10*). Discussion and debate about the importance of each risk element to answer the 2 questions resulted in a consensus ranking of risk elements for each question after 2 rounds of ranking all elements. Subsequently, when viruses were evaluated by subject-matter experts (SMEs), their task was to consider a virus solely within the definition of the individual risk element they were scoring. SMEs scored a specifically selected virus of interest; only data related to this strain were considered to avoid confusion over potentially significant strain differences. Each evaluation was conducted in the context of data available at the time of the evaluation. Multiple SMEs scored each risk element, but the maximum number of risk elements scored by any one SME evaluating a virus was set at 3 in an attempt to maintain a high level of expertise, assuming that most SMEs are not experts across all the technical areas represented by the various risk elements. Limiting the number of elements any SME scores also reduced the burden on any one SME, which can potentially shorten the time to evaluate a given virus and removed the possibility of potential bias introduced by an SME scoring most or all elements.

**Figure 1:**
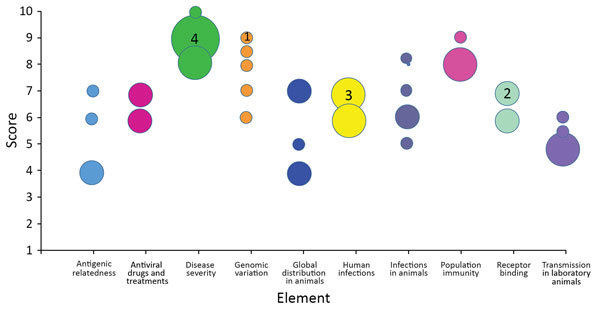
Individual subject-matter expert point scores by element for the May 2017 scoring of influenza A(H7N9) virus, A/Hong Kong/125/2017, based on risk element definitions. Circles indicate individual point scores; circle sizes (examples indicated by a number inside) correspond to the frequency of each point score.

The SMEs provided a point estimate based on a standardized scale of 1–10 using the definition and criteria of the risk element. For each element, the lowest risk score possible is 1, and the highest risk score possible is 10. The SMEs also provided an upper and lower bound of acceptability in scores they would consider reasonable from other experts, assuming the same knowledge base regarding the risk element. In addition, a justification was solicited that summarized the critical basis contributing to the SME’s point estimate, as well as his or her judgment of confidence in the quality of the data. Scoring was collected in 2 phases. In the initial phase, preliminary data were reviewed, and variation in the individual risk element scores was noted. A wide range of point estimate scores for a particular element potentially indicated that SMEs operated from a different knowledge base, such as when unpublished data were available to only a select few or alternatively indicated that few data were available to use in generating scores. In the second phase, SMEs were presented with a summary of the preliminary data for their element(s). In instances with a wide range of scores for a given element, the justification information was anonymized and redistributed to all the SMEs who scored that particular element, with the invitation to reconsider or confirm their initial score (Figure 1). Results for the IRAT average point scores were used to calculate an overall virus risk score for each of the 2 risk questions. Generally, virus scores of 1–3 were considered a low potential risk by the IRAT; scores of 4–7 were moderate; and scores of 8–10 were potential high risk. Scores at the boundaries of those ranges are described by a combination term such as moderate-high for a score of 7.5.

### Establishment of a Point of Reference

As a point of reference for low-risk viruses with respect to both IRAT questions, potential risk for emergence and risk for potential impact, SMEs evaluated the North America avian influenza A(H1N1) virus, A/duck/New York/1996. As expected, this virus received low risk scores from the SMEs; the summary average risk score was 2.3 (i.e., low risk) to achieve sustained human-to-human transmission. Similarly, the average risk score for the virus to substantially impact public health if it were to achieve sustained human-to-human transmission was 2.4 (low risk).

## Results

**Figure 2:**
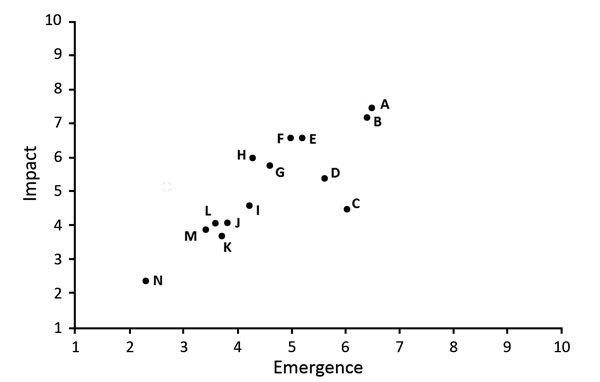
Comparison of average emergence and impact scores for 14 animal-origin influenza viruses using the Influenza Risk Assessment Tool. Circle represents each virus: A, H7N9 A/Hong Kong/125/2017; B, H7N9 A/Shanghai/02/2013; C, H3N2 variant A/Indiana/08/2011; D, H9N2 G1 lineage A/Bangladesh/0994/2011; E, H5N1 clade 1 A/Vietnam/1203/2004; F, H5N6 A/Yunnan/14564/2015-like; G, H7N7 A/Netherlands/2019/2003; H, H10N8 A/Jiangxi-Donghu/346/2013; I, H5N8 A/gyrfalcon/Washington/41088/2014; J, H5N2 A/Northern pintail/Washington/40964/2014; K, H3N2 A/canine/Illinois/12191/2015; L, H5N1 A/American green-winged teal/Washington/1957050/2014; M, H7N8 A/turkey/Indiana/1573-2/2016; N, H1N1 A/duck/New York/1996. Additional information about virus scores and individual viruses is available at https://www.cdc.gov/flu/pandemic-resources/monitoring/irat-virus-summaries.htm.

During 2011–2017, SMEs evaluated 14 animal-origin influenza viruses using the IRAT. The emergence and impact scores are plotted for each virus (Figure 2). Of the viruses scored thus far by IRAT, avian influenza A(H7N9) A/Hong Kong/125/2017 ranked highest for potential risk. Other viruses, such as A/Indiana/08/2011, an influenza A(H3N2) variant (H3N2v), had a similar score for risk for emergence similar to that of A/Hong Kong/125/2017 but a much lower estimated risk for potential impact.

### Influenza A(H7N9) and the IRAT

On March 31, 2013, the China Health and Family Planning Commission notified the World Health Organization (WHO) of 3 cases of human infection with influenza A(H7N9) (*14*). Three viruses were isolated and analyzed at the China WHO Collaborating Center and the complete viral genome sequences deposited in a publicly accessible influenza database. After these reports, CDC used the IRAT to assist the US Department of Health and Human Services’ Biomedical Advanced Research and Development Authority with the overall prepandemic risk assessment of these viruses.

Although laboratories had begun the animal transmission challenge work, study results were not available. Hence, the IRAT risk element of transmissibility in animal models lacked data. This element is ranked as the second most important and thus carries a high weight in the computation of a final score for the IRAT emergence question. Therefore, it was necessary to assign a score for this element without data. Point scores for the other 9 elements were gathered and used to populate the IRAT to generate a partial risk score.

Although information about the outcome of laboratory animal transmission studies was scarce, previous observations showed significant correlation between other IRAT risk elements (receptor-binding properties, genomic variation, and human infections) and this element. A moderate score for this element extrapolated from other elements would greatly improve the ability to compare this new virus with other viruses evaluated previously with the IRAT. Based on the evidence of increased α2,6 receptor binding, the presence of L226 in the HA receptor binding pocket and the ability to infect humans, this element was assigned a score of 7 in the moderate risk category (range 4–7). Uncertainty was captured by assigning the risk element transmissibility in animal models a score of 1, 7, or 10 (Table 1). Using these 3 possible scores for this risk element, the summary risk score for the emergence question would be 5.2, 6.4, or 7.0, respectively. Assigning these same scores to this risk element to calculate the impact score, the summary risk scores would be 7.1, 7.4, or 7.5, respectively (Table 2). The much greater range in emergence (1.7) than impact (0.3) score is understandable when the relative weight assigned to this risk element is considered in the 2 different scenarios.

**Table 1:** Emergence question IRAT score calculation for 2 similar influenza A(H7N9) viruses, A/Shanghai/2/2013 and A/Anhui/1/2013, with data missing on the risk element “transmission in laboratory animals” during scoring of 2013 outbreak in China*

Element	W	Transmission in laboratory animals

R = 1†			R = 7‡			R = 10§	
R	W × R	R	W × R	R	W × R
Human infections	0.2929	5.00	1.46			1.46			1.46
Transmission in lab animals	0.1929	1.00	0.19		7.00	1.35		10.00	1.93
Receptor binding	0.1429	6.70	0.96			0.96			0.96
Population immunity	0.1096	9.00	0.99			0.99			0.99
Infections in animals	0.0846	6.00	0.51			0.51			0.51
Genomic variation	0.0646	8.60	0.56			0.56			0.56
Antigenic relatedness	0.0479	6.00	0.29			0.29			0.29
Global distribution in animals	0.0336	1.00	0.03			0.03			0.03
Disease severity and pathogenesis	0.0211	9.00	0.19			0.19			0.19
Antiviral drug and treatment options	0.0100	5.40	0.05			0.05			0.05
Total	1.0001		5.24			6.40			6.98

*IRAT, Influenza Risk Assessment Tool; R, average risk point score; W, weight. †Substituting the lowest possible risk score (1) to calculate summary IRAT score. ‡Substituting a moderate risk score (7) to calculate summary IRAT score.  §Substituting the highest possible risk score (10) to calculate summary IRAT score.

**Table 2:** Impact question IRAT score calculation for 2 similar influenza A(H7N9) viruses, A/Shanghai/2/2013 and A/Anhui/1/2013, with data missing on the risk element “transmission in laboratory animals” during scoring of 2013 outbreak in China*

Element	W	Transmission in laboratory animals

R = 1†			R = 7‡			R = 10§	
R	W × R	R	W × R	R	W × R
Disease severity and pathogenesis	0.2929	9.00	2.64			2.64			2.64
Population immunity	0.1929	9.00	1.74			1.74			1.74
Human infections	0.1429	5.00	0.71			0.71			0.71
Antiviral drug and treatment options	0.1096	5.40	0.59			0.59			0.59
Antigenic relatedness	0.0846	6.00	0.51			0.51			0.51
Receptor binding	0.0646	6.70	0.43			0.43			0.43
Genomic variation	0.0479	8.60	0.41			0.41			0.41
Transmission in lab animals	0.0336	1.00	0.03		7.00	0.24		10.00	0.34
Global distribution in animals	0.0211	1.00	0.02			0.02			0.02
Infection in animals	0.0100	6.00	0.06			0.06			0.06
Total	1.0001		7.14			7.35			7.45

*IRAT, Influenza Risk Assessment Tool; R, average risk point score; W, weight.  †Substituting the lowest possible risk score (1) to calculate summary IRAT score. ‡Substituting a moderate risk score (7) to calculate summary IRAT score.  §Substituting the highest possible risk score (10) to calculate summary IRAT score.

Only minimal data were available for 2 other elements (global distribution in animals and infections in animals) in April 2013. For the purposes of the risk scoring, we gave the global distribution in animals element a score of 1 because this virus had been identified in only a few live-bird markets in China. Because of the lack of information, confidence scores were low for this element. SMEs gave infection in animals a higher risk (mean 6, moderate risk) and confidence scores probably because of other H7N9 viruses associated with avian species. Because these elements carry less weight in risk scoring for both questions, they did not heavily affect the final score. In general, the SMEs agreed about risk scores for these elements.

The uncertainty and the data gaps, particularly for the transmissibility in the animal models element, were presented to decision makers and discussed. Particular attention was given to explain the range of scoring generated about the emergence question. However, the SMEs agreed that the impact score was less influenced by the missing information and the risk score did not significantly affect the final summary score.

In May 2013, 1 month after the initial assessment, information became available to inform the risk element transmissibility in animal models. More information was available for all other elements as well. The viruses were rescored in May 2013. The resulting average summary risk score for the 2 similar influenza A(H7N9) viruses (A/Anhui/1/2013 and A/Shanghai/2/2013) was 6.4 for the emergence risk and 7.2 for impact on public health if this virus gains the ability to transmit from person to person. SMEs reported a higher level of confidence in their risk scores at this time, although most element risk scores did not change appreciably. Since May 2013, these viruses have been scored annually in 2014, 2015, 2016, and again in early 2017, with little to no change in scoring, but with higher levels of confidence in individual scores each year.

### Adaptation of the IRAT to Assess Influenza A(H5N1) Viruses

In 2014, the IRAT was used to compare several influenza A(H5N1) viruses that circulated during 2011–2014. Use of the IRAT is predicated on the assumption that each risk element will independently assess some aspect inherent in or associated with the various viruses included in the assessment. Based on available information, 5 of the risk elements would have had virtually the same score for all the H5N1 viruses. Although these 5 risk elements are useful for discriminating among other viruses, when comparing H5N1 viruses, sufficient information is lacking to enable the distinctions among the viruses necessary for the IRAT. These 5 elements (disease severity, population immunity, antiviral treatment susceptibility, receptor-binding properties, and transmissibility in animal models) were therefore removed from the IRAT scoring.

To use the IRAT to compare these viruses with each other, we tailored specific questions for this effort. Two questions were generated that related specifically to prepandemic mitigation of the impact these viruses could have on public health. The risk assessment focused on 2 questions about the risk element of antigenic relationship and availability of vaccines:

1. Considering the vaccine antigens that are in the US Strategic National Stockpile or are currently being generated for this purpose, what H5N1 viruses pose the greatest potential risk to public health?
2. Considering what CVVs are available or in development, what H5N1 viruses pose the greatest potential risk to public health?

To answer these 2 questions, SMEs scored the element of antigenic relatedness twice, in relationship to 1) a currently available WHO CVV or 2) antigens already prepared and stockpiled. Stockpiled antigen would be more quickly available for use than antigen in early development as a CVV.

Ten H5N1 clades were considered to be circulating during 2011–2014. SMEs scored these 10 using the IRAT based on information available for 5 elements that could be used to distinguish between these related viruses. SMEs were asked to provide a risk score for 1) human infections, 2) antigenic relationship of the viruses, 3) global distribution in animals, 4) infections in animals, and 5) genomic variation. The elements are listed in order of importance (i.e., the most heavily weighted element is human infections, and the next most important is the antigenic relatedness).

Initial SME scores for the 5 elements were averaged and presented to the same SMEs, and consensus on the final scores was reached through discussion. These risk scores were then multiplied by the appropriate weighting factor to generate summary risk scores. Because this risk assessment comprises only 5 elements, weights were apportioned on the basis of 5 elements rather than on the standard 10 elements. The IRAT definitions for the elements remained the same.

When scoring the 10 H5N1 viruses for antigenic relatedness, the SMEs based their first risk score on knowledge of currently available WHO CVVs and applied the IRAT definition of antigenic relatedness. The same SMEs then provided a second risk score for antigenic relatedness to US stockpiled antigens when considering the same 10 clades. In some instances, the average risk scores for this element differed. In each case, the average risk score for this element was multiplied by 0.2567, providing 2 possible summary risk scores for each virus (Tables 3, 4).

**Table 3:** Product of average risk point scores multiplied by weight for each of the 10 influenza A(H5N1) clades for the IRAT when the antigenic relatedness score is based on the virus’ relatedness to a CVV*

Element	Clade									
	1.1.1	1.1.2	2.1.3.2a	2.2.1	2.2.1.1	2.3.2.1a	2.3.2.1b	2.3.2.1c	2.3.4.2	7.2
Human infections	1.96	3.06	2.28	2.28	0.91	1.96	1.05	1.83	1.83	0.59
Antigenic relatedness to CVV	0.59	0.69	0.59	0.59	1.54	0.59	0.59	0.59	0.69	2.05
Global distribution in animals	0.63	0.78	0.78	0.47	0.47	0.94	0.89	1.21	0.74	0.67
Infection in animals	0.45	0.63	0.63	0.66	0.27	0.57	0.48	0.72	0.51	0.42
Genomic variation	0.12	0.25	0.13	0.13	0.18	0.16	0.12	0.25	0.11	0.19
Total	3.75	5.42	4.42	4.13	3.27	4.22	3.13	4.60	3.87	3.93

*Weight for human infections = 0.4567; weight for antigenic relatedness to CVV = 0.2567; weight for global distribution in animals = 0.1567; weight for infections in animals = 0.09; weight for genomic variation = 0.04. CVV, candidate vaccine virus; IRAT, Influenza Risk Assessment Tool.

**Table 4:** Product of average risk point scores multiplied by weight for each of the 10 influenza A(H5N1) clades for the IRAT when the antigenic relatedness score is based on the virus’ relatedness to a US stockpiled antigen*

Element	Clade									
	1.1.1	1.1.2	2.1.3.2a	2.2.1	2.2.1.1	2.3.2.1a	2.3.2.1b	2.3.2.1c	2.3.4.2	7.2
Human infections	1.96	3.06	2.28	2.28	0.91	1.96	1.05	1.83	1.83	0.59
Antigenic relatedness to stockpiled antigen	0.77	1.21	1.28	0.69	2.05	1.98	1.98	1.98	1.62	2.05
Global distribution in animals	0.63	0.78	0.78	0.47	0.47	0.94	0.89	1.21	0.74	0.67
Infection in animals	0.45	0.63	0.63	0.66	0.27	0.57	0.48	0.72	0.51	0.42
Genomic variation	0.12	0.25	0.13	0.13	0.18	0.16	0.12	0.25	0.11	0.19
Total	3.93	5.94	5.11	4.23	3.78	5.61	4.52	5.99	4.80	3.93

*Weight for human infections = 0.4567; weight for antigenic relatedness to candidate vaccine virus = 0.2567; weight for global distribution in animals = 0.1567; weight for infections in animals = 0.09; weight for genomic variation = 0.04. IRAT, Influenza Risk Assessment Tool.

When SMEs considered risk scores associated with antigenic relatedness to CVVs, clade 1.1.2 was the only virus clade that scored >5.0. When considering summary risk scores when the antigenic relatedness element was based on already stockpiled antigens, SMEs scored 4 virus clades >5.0: clade 1.1.2, clade 2.1.3.2a, clade 2.3.2.1a, and clade 2.3.2.1c (Table 5).

**Table 5:** Summary of scoring of the 7 highest-scoring influenza A(H5N1) virus clades related to antigen in the US SNS and to nearest related CVV*

Clade	Antigenic relatedness*	
	To SNS	To CVV
2.3.2.1c	5.99	4.60
1.1.2	5.94	5.42
2.3.2.1a	5.61	4.22
2.1.3.2a	5.11	4.42
2.3.4.2	4.80	3.87
2.3.2.1b	4.52	3.13
2.2.1	4.23	4.13

*Relatedness based on hemagglutinin inhibition testing, candidate virus vaccine; SNS, Strategic National Stockpile.

Ultimately, the US Department of Health and Human Services’ Biomedical Advanced Research and Development Authority decided to base the antigen to add to the US stockpile on influenza A(H5N1) clade 2.3.2.1a. This clade did not score the highest through the IRAT, but additional information, such as production deadlines, availability of the CVVs, and contractual obligations, also was considered before a final decision was reached, reinforcing that the IRAT is just 1 input for decision makers.

## Discussion

The objective of the IRAT development was to assist decision makers in pandemic planning by creating a tool that facilitates the assessment of influenza A viruses not circulating in humans but potentially posing a pandemic risk. A common misconception is that the IRAT is a prediction tool to identify the next likely pandemic virus; however, that is neither the intent nor within the capability of the IRAT. Without a complete understanding of all the mechanisms and factors associated with the emergence of a pandemic virus, let alone the plausibility of detecting and characterizing the immediate precursor of the next pandemic influenza virus, prediction is not possible at this time. However, on the basis of 10 individual risk elements weighted specifically in relationship to their importance in answering specific risk questions, the IRAT process evaluates viruses systematically. This assessment enables comparison of different viruses when prioritization decisions must be made.

The IRAT development objective was fulfilled in 3 important ways. First, the IRAT provides a systematic procedure and framework for acquiring, analyzing, and combining information on multiple attributes of influenza A viruses deemed important to the consideration and communication of pandemic risk by influenza SMEs. The IRAT simplifies interpretation of multiple complex data elements but requires the interpretation of complex data by SMEs within their respective areas of expertise to generate an overall assessment of the perceived pandemic risk. Second, the IRAT has shown the requisite flexibility required to deal with practical issues of characterizing newly emerging influenza viruses, such as lack of data within specific risk elements. As in the initial evaluations of influenza A(H7N9), to address missing data regarding a critical risk element, a range of scores for that element was used to generate a range of possible summary risk scores that was easily communicated to decision makers. Third, the IRAT supports CDC’s Pandemic Preparedness and Response Framework by summarizing information to assist in prepandemic decisions (*13*).

Other initiatives addressing pandemic influenza risk assessment have taken an approach similar to IRAT or used a modification of epidemiologic risk modeling. WHO’s Global Influenza Program has recently introduced the Tool for Influenza Pandemic Risk Assessment (TIPRA) (*15*) to supplement its Pandemic Influenza Risk Management guideline (*16*). Although TIPRA uses the same decision analysis approach as IRAT, some subtle and some more major differences make the TIPRA unique. Risk questions similar to the IRAT are used, but fewer individual risk elements are used along with different definitions. In addition, a gateway question of evidence for population immunity dictates whether use of the TIPRA is indicated. An alternative approach has been taken by the FLURISK project (*17*), an activity funded by the European Food Safety Authority. By combining an estimate of human–livestock contact intensity with influenza strain–specific outbreak information and the virus’ estimated capability to cause human infection, a quantitative risk for >1 human infections is calculated. Pandemic risk is therefore not specifically addressed in this model, but rather the risk for an influenza A virus to make the species jump into humans, a prerequisite of a pandemic, is estimated (*18*).

As research progresses into influenza virus mechanisms of transmission and adaptation to mammalian hosts, particularly in relation to humans, more risk elements for use in IRAT may be identified or existing risk elements may be modified and redefined. In a recent review of pandemic influenza risk assessment, the review authors contended that assessment of influenza pandemic risk should include 3 specific viral factors: HA receptor binding specificity, HA pH of activation, and polymerase complex efficiency (*19*). IRAT addresses receptor binding specificity directly but does not specifically incorporate the other 2 factors. Consideration of these, as well as other sources of data related to answering the IRAT risk questions, will be investigated for potential inclusion in the IRAT. Thus, the IRAT is a carefully defined tool that provides standardized risk assessment scores and a flexible framework that can be modified for special cases and as additional information becomes available.
